# A Patient Developed Painful Muscle Cramps due to Overeating Mangos

**DOI:** 10.1155/2012/742125

**Published:** 2012-10-04

**Authors:** Kazuo Abe

**Affiliations:** Clinical Research Center, Osaka Health Science University, 1-9-27 Tenma, Kita-Ku, Osaka 530-0043, Japan

## Abstract

A 79-year-old woman had a habit to eat a mango every night before sleep and experienced muscle cramps during sleep. Her muscle cramps may be resulted from potassium overload due to overeating mangos.

## 1. Introduction

Mangiferin is a polyphenolic compound abundant in the stem bark of *Mangifera indica* (mango) with antioxidant and anti-inflammatory properties in different experimental settings [1]. These experimental settings reports have attracted popular interesting and many tend to eat more mangos to prevent ischemic heart and brain diseases. However, overeating mango may not be good for health. We experienced a woman developing every night muscle cramps after overeating mangos.

## 2. Case Report

A 79-year-old woman presented with everyday history of muscle cramps and parenthesis during sleep. She reported receiving no medications and specifically reported not using thiazide diuretics before these episodes. She had a blood pressure of 120/64 mm Hg, a respiratory rate of 19 breaths per minute. On admission in the morning, laboratory investigation revealed a serum calcium level of 2.5 mmol per liter (normal range, from 2.2 to 2.6), a potassium level of 5.5 mmol per liter (normal range, from 3.5 to 5.0), and a magnesium level of 0.9 mmol per liter (normal range, from 0.8 to 1.2). On inquiring, she advised us that she had eaten a mango every night from a month before for good sleep. A diagnosis of muscle cramps due to hyperpotassium was made. She stopped eating mango fruits and showed normal potassium level of 4.8 mmol per liter on the next admission. She had not experienced muscle cramps since then.

## 3. Discussion

Mango becomes popular food because of its effect for decrement of inflammation and of oxidative damage and is a potassium rich food with 170 mg potassium per 100 g weight. Potassium is one of the most important micronutrients required by the human body. It serves various functions including firing of neurons, maintenance of heart rhythm to the contraction of muscles. However, overeating of mangoes may result in excessive levels of potassium and develop hyperkalemia. Hyperkalemia has been known in patients with renal failure and luxury potassium uptake. Hyperkalemia is also known to develop painful muscle cramps. A painful muscle cramp is an involuntarily and forcibly contracted muscle that does not relax. When we use the muscles that can be controlled voluntarily, such as those of our arms and legs, they alternately contract and relax as we move our limbs [2]. A muscle that involuntarily contracts is in a spasm. If the spasm is forceful and sustained, it becomes a cramp. Painful muscle cramps often cause a visible or palpable hardening of the involved muscle. Muscle cramps are extremely common. Almost everyone experiences a cramp. Muscle cramps can be categorized into four major types. These include true cramps, tetany, contractures, and dystonic cramps. True cramps involve part or all of a single muscle or a group of muscles that generally act together, such as the muscles that flex several adjacent fingers. Abnormal blood levels of electrolytes, such as calcium, magnesium, or even potassium, can develop muscle cramps. Although low potassium blood levels occasionally cause true muscle cramps, high potassium blood levels also cause muscle cramps.

Although nocturnal muscle cramps increase its frequency along with aging, our patient had never experienced nocturnal muscle cramps. Recently, painful muscle cramps have been reported in families with dominantly inherited myotonic disease potassium-aggravated myotonia [3–5]. Our patient denied family history. Thus, painful muscle cramps in our patient were possibly due to high blood potassium level after overeating mango. The observation that her painful muscle cramps stopped after cessation to eat mango at night supports our conclusion.
